# Effectiveness of nutrition and dietary interventions for people with serious mental illness: systematic review and meta‐analysis

**DOI:** 10.5694/mja2.51680

**Published:** 2022-10-02

**Authors:** Tetyana Rocks, Scott B Teasdale, Caitlin Fehily, Claire Young, Gina Howland, Blair Kelly, Samantha Dawson, Felice Jacka, James A Dunbar, Adrienne O’Neil

**Affiliations:** ^1^ Institute for Mental and Physical Health and Clinical Translation (IMPACT) Deakin University Melbourne VIC; ^2^ University of New South Wales Sydney NSW; ^3^ Mindgardens Neuroscience Network Sydney NSW; ^4^ University of Newcastle Newcastle NSW; ^5^ Institute for Mental and Physical Health and Clinical Translation (IMPACT) Deakin University Geelong VIC; ^6^ Deakin University Melbourne VIC; ^7^ Murdoch Children’s Research Institute Melbourne VIC; ^8^ Deakin University Warrnambool VIC; ^9^ Deakin University Geelong VIC

**Keywords:** Schizophrenia spectrum and other psychotic disorders, Mood disorders, Diet, Nutrition, Metabolic diseases

## Abstract

**Objective:**

To review recent published trials of nutrition and dietary interventions for people with serious mental illness; to assess their effectiveness in improving metabolic syndrome risk factors.

**Study design:**

Systematic review and meta‐analysis of randomised and non‐randomised controlled trials of interventions with a nutrition/diet‐related component delivered to people with serious mental illness, published 1 January 2010 – 6 September 2021. Primary outcomes were weight, body mass index (BMI), and waist circumference. Secondary outcomes were total serum cholesterol, low‐density lipoprotein (LDL) and high‐density lipoprotein (HDL) cholesterol, triglyceride, and blood glucose levels.

**Data sources:**

MEDLINE, EMBASE, PsycINFO, CINAHL, and CENTRAL databases. In addition, reference lists of relevant publications were examined for further additional studies.

**Data synthesis:**

Twenty‐five studies encompassing 26 intervention arms were included in our analysis. Eight studies were at low or some risk of bias, seventeen were deemed to be at high risk. Eight of seventeen intervention arms found statistically significant intervention effects on weight, ten of 24 on BMI, and seven of seventeen on waist circumference. The pooled effects of nutrition interventions on metabolic syndrome risk factors were statistically non‐significant. However, we identified small size effects on weight for interventions delivered by dietitians (five studies; 262 intervention, 258 control participants; standardised mean difference [SMD], –0.28; 95% CI, –0.51 to –0.04) and interventions consisting of individual sessions only (three studies; 141 intervention, 134 control participants; SMD, –0.30; 95% CI, –0.54 to –0.06).

**Conclusions:**

We found only limited evidence for nutrition interventions improving metabolic syndrome risk factors in people with serious mental illness. However, they may be more effective when delivered on an individual basis or by dietitians.

**PROSPERO registration:**

CRD42021235979 (prospective).

Compared with the general population, people with serious mental illness are at significantly greater risk of physical health problems, including metabolic syndrome, which comprises obesity, high blood pressure, dyslipidaemia, and hyperglycaemia.^1^ This cluster of risk factors is associated with greater risk of cardiovascular disease and premature mortality among people with serious mental illness.^2^

This poorer physical health is multifactorial in origin, stemming from fragmented health services and diagnostic overshadowing,^3^ illness characteristics and medication side effects,^4^, ^5^ greater use of tobacco,^6^ alcohol, and other substances,^7^ excessive and poor quality dietary intake,^8^, ^9^ high levels of sedentary behaviour,^10^ and low levels of cardio‐respiratory fitness.^11^ In response, the World Health Organization has published management guidelines,^12^ and a *Lancet Psychiatry* Commission was established to develop strategies for protecting the physical health of people with mental illness.^13^ Both documents highlight dietary intervention as a critical element.

Targeting diet for preventing cardiovascular disease in general is supported by extensive research.^14^ However, people living with serious mental illness experience specific additional challenges that may require the adjustment of dietary interventions, including increased appetite as a side effect of psychotropic medication (especially second generation antipsychotics),^15^ less sensitive neural reward systems and poor cognitive control,^16^ high rates of disordered eating behaviour (eg, binge eating, emotional eating),^17^ high rates of food insecurity,^18^ and lack of motivation to engage with treatment and implement dietary recommendations.^19^

A 2020 systematic review of published randomised controlled trials (to 2017) found that dietary interventions were effective for improving weight, body mass index (BMI), waist circumference, and blood glucose levels in people with schizophrenia, related psychoses, or bipolar disorder.^20^ Despite considerable differences between intervention elements and their effects, interventions delivered by a dietitian or delivered during the early stages of illness and antipsychotic therapy were identified as most effective. As the review did not include interventions for people with clinical depression, the Being Equally Well (www.vu.edu.au/mitchell‐institute/health‐systems‐change/being‐equally‐well‐roadmap) expert working group recommended an update. We have therefore reviewed recently published dietary intervention trials that aimed to reduce metabolic syndrome risk in people with serious mental illness.

## Methods

This systematic review and meta‐analysis was pre‐registered with the PROSPERO database (CRD42021235979; 10 April 2021) and is reported in accordance with the PRISMA guidelines.^21^

### Search strategy

We searched MEDLINE Complete, APA PsycInfo, and CINAHL Complete (via EBSCOhost), EMBASE (at Embase.com), and CENTRAL (via the Cochrane Library) for studies published since 1 January 2010. Our searches on 26 March 2021 and 6 September 2021 used a comprehensive strategy developed by a health librarian in consultation with the review team (Supporting Information, tables 1–5). We examined the reference lists of identified publications for further relevant items.

We included studies in our analysis that evaluated nutrition‐related interventions (as sole interventions or as part of broader interventions) for people with a serious mental illness (major depressive disorder, bipolar affective disorder, schizophrenia and related psychoses), had a primary aim of improving body mass, blood pressure, or metabolic biochemistry measures, included a control group (standard mental health care), and were published in English (Supporting Information, table 6). We included full original research articles and brief reports, but not review articles, letters to the editor, dissertations, conference abstracts, or other grey literature.

After removing duplicates, five reviewers (TR, CF, CY, GH, BK) screened the titles and abstracts of the identified records, and then the full text of relevant articles. Disagreements about including studies were resolved by consensus, and reasons for excluding articles during the full‐text screening phase recorded.

### Data extraction

Data were independently extracted from each eligible article by two of four reviewers (TR, CF, CY, GH) and disagreements resolved by consensus. We extracted data for year and country of study; primary and secondary aims; inclusion and exclusion criteria; population description and diagnoses, including numbers in intervention and control groups at each stage; medications; intervention description, including length, type (diet‐only or diet/nutrition program as part of a broader intervention), delivery method and personnel, and components; control group conditions; baseline and post‐intervention metabolic syndrome indicators; and limitations.

### Outcomes

The outcomes were physical parameters relevant to the metabolic syndrome. Primary outcomes were weight, BMI, and waist circumference. Secondary outcomes were total serum cholesterol, low‐density lipoprotein (LDL) and high‐density lipoprotein (HDL) cholesterol, and triglyceride levels, and blood glucose levels.

### Bias risk

We assessed risk of bias with the modified Cochrane risk of bias in randomised controlled trials (RoB2)^22^ and risk of bias in cluster randomised trials tools (RoB2 Cluster),^23^ and with the risk of bias in non‐randomised studies of interventions tool (ROBINS‐I).^24^ For randomised controlled and cluster randomised trials, overall risk of bias was deemed high if risk was high in at least one domain; for non‐randomised interventions, the risk of bias was deemed serious when a serious risk was determined in at least one domain. For randomised controlled and cluster randomised trials, the “effect of adhering to intervention” was used for the “bias due to deviations from intended interventions” domain; correspondingly, the “effect of starting and adhering to intervention” was used for non‐randomised studies. Risk of bias was assessed by four reviewers (TR, CF, CY, GH) and disagreements were resolved by consensus. Assessments were grouped by included study type with figures created with Risk‐of‐bias VISualization (robvis).^25^

### Certainty of evidence

Certainty of evidence for each outcome was assessed by two reviewers (SLD, TR) using the Grading of Recommendations Assessment, Development and Evaluation (GRADE) framework.^26^ The GRADE assessment comprises four dimensions: overall risk of bias, inconsistency, indirectness, and imprecision. Certainty for an outcome was reduced if based on studies with serious bias according to the RoB2, RoB2 Cluster, and ROBINS‐I assessments, or if the outcome was derived from highly heterogeneous studies. Inconsistency was evaluated as heterogeneity in study design with respect to participants (inclusion and exclusion criteria), intervention, control group definition, and outcomes, and was quantified by estimating *I* ^2^ for each outcome in the meta‐analysis. Certainty was reduced if the outcome was an indirect or secondary outcome of a study; that is, according to whether the study was designed to measure the outcome as the main or a primary study outcome. Imprecision was reduced if study sample sizes were small and confidence intervals consequently wide, or if there was marked variation in dietary intervention and adherence.

### Data synthesis

In our primary meta‐analysis, we assessed the effects of dietary interventions on metabolic outcomes (compared with the comparator management). We report standardised mean differences (SMDs) for continuous outcome measures, using post–post means with 95% confidence intervals (CIs). SMD effect size was deemed small at 0.2, medium at 0.5, and large at 0.8. Analyses were performed in Review Manager (RevMan) 5.4 (Cochrane Collaboration). Because of heterogeneity in study characteristics, we applied a random effects model. Heterogeneity was quantified with the *I* ^2^ statistic. Publication bias was assessed by inspection of funnel plots. Subgroup analyses assessed the effect of mode of delivery (individual, group, mixed) and of the professional delivering the intervention (dietitian, other).

## Results

Our systematic database and citation search identified 3350 unique titles; we excluded 3233 after title and abstract screening, and 92 after full text screening. We included 25 publications in this review (Box 1).^27^, ^28^, ^29^, ^30^, ^31^, ^32^, ^33^, ^34^, ^35^, ^36^, ^37^, ^38^, ^39^, ^40^, ^41^, ^42^, ^43^, ^44^, ^45^, ^46^, ^47^, ^48^, ^49^, ^50^, ^51^

Box 1 **PRISMA flow diagram for selection of publications for inclusion in our analysis**

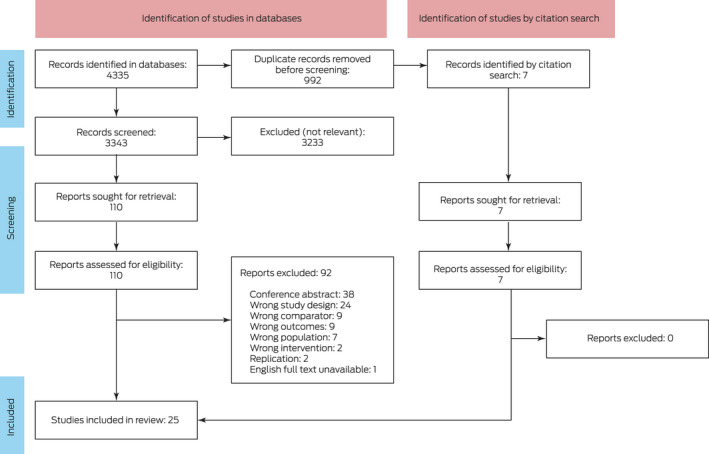

### Study characteristics

Twenty studies were randomised controlled trials,^27^, ^28^, ^29^, ^31^, ^32^, ^33^, ^34^, ^35^, ^36^, ^37^, ^38^, ^39^, ^40^, ^41^, ^42^, ^44^, ^46^, ^47^, ^49^, ^50^ three were cluster randomised controlled trials,^43^, ^48^, ^51^ and two were non‐randomised controlled trials.^30^, ^45^ Twenty‐four studies included intervention and control arms.^27^, ^28^, ^29^, ^30^, ^31^, ^32^, ^33^, ^34^, ^35^, ^36^, ^37^, ^38^, ^39^, ^40^, ^41^, ^42^, ^43^, ^44^, ^45^, ^46^, ^47^, ^48^, ^50^, ^51^ One study^49^ was a three‐arm trial (two intervention arms and one control group); for our review and analysis, we separately compared each intervention arm with the control arm, yielding a total of 26 comparisons of intervention arms with control arms (Box 2).

Box 2 **Characteristics of the published trials included in our systematic review and meta‐analysis**

**Table:** 

Study	Participants (women); age (years), mean (SD)			Intervention group			Control group	
	Control	Intervention	Diagnoses	Type,* length, delivery mode, personnel	Description	Adherence/ attendance	Description	Primary outcomes
Attux 2013^27^ (Brazil; RCT)	81 (33) 36.2 (9.9)	79 (31) 38.3 (10.7)	SCH, other psychosis	Multiple 12 weeks Group Dietitian	12 sessions: introduction, four sessions on diet/nutrition; three sessions on physical activity; sessions on self‐esteem, motivation, management of anxiety; session with relatives; wrap‐up	Mean, 9.1 (SD, 3.5) sessions 49 (72%) attended at least eight sessions.	Standard care	Mean weight change: 3 months: intervention, –0.48 kg (95% CI, –0.65 to +1.13); control, +0.48 kg (95% CI, 0.13–0.83; *P* = 0.06). 6 months: intervention, –1.15 kg (95% CI, –2.11 to +0.19); control, +0.5 kg (95% CI, –0.42 to +1.42; *P* = 0.017).
Brown 2011^28^ (USA; RCT)	68 (54) 44.6 (10.9)^†^	68 (54) 44.6 (10.9)^†^	SCH, BPD, other psychosis	Multiple 52 weeks Mixed Dietitian	3‐month intense phase (weekly 3 h sessions on nutrition, physical activity, goal setting); 3‐month maintenance phase (monthly meetings, weekly phone calls); 6‐month intermittent support phase	21/68 (31%) discontinued within 6 months; no information for 12 months	Standard care, including voluntary day program	Mean weight change (completers only): Intervention, –2 kg; control, –0.4 kg (*P* = 0.005).
Cordes 2014^29^ (Germany; RCT)	38 (11) 35.8 (10.9)	36 (21) 38.2 (11.2)	SCH	Multiple 24 weeks Group Dietitian	12 fortnightly sessions. Four modules: assessment of eating, physical activity; healthy isocaloric diet; practical lessons in skill building; behavioural techniques, stress management, coping strategies.	25/36 (64%) intervention group discontinued within 24 weeks	Standard care	Weight change (24 weeks): No difference between groups. Waist circumference (48 weeks): intervention, +4.6 cm (SD, 8.3); control, +10.1 cm (SD, 7.3) (*P* = 0.019). Fasting plasma glucose (48 weeks): smaller increase in intervention group (*P* = 0.031)
Curtis 2016^30^ (Australia; nRCT)	12 (2) 21.7 (1.9)	16 (9) 20.0 (2.3)	SCH, BPD, MDD	Multiple 12 weeks Mixed Dietitian	Individualised program with three components: health coaching, weekly dietetic support (education [weight management, labels, food quality], and practical skills [shopping, cooking]), supervised physical activity sessions.	Mean, 8 diet sessions (range, 5–10); 11 physical activity sessions (range, 3–25)	Standard care	Mean weight change (12 weeks): Intervention, +1.8 kg (95% CI, −0.4 to +2.8); control, 7.8 kg (95% CI, 4.8–10.7; *P* < 0.001).
Daumit 2013^31^ (USA, RCT)	147 (72) 44.1 (11.0)	144 (74) 46.6 (11.5)	SCH, BPD, MDD, other^‡^	Multiple 78 weeks Mixed Health educator/ coach	1–6‐month intensive phase: group classes (weight management 1/week; physical activity, 3/week); monthly individual visits. 7–18 month maintenance phase: group classes (weight management 1/month; physical activity 1/week); monthly individual visits. Weight management topics: tracking; food choices; portions, snacking; fruit, vegetables. Behavioural techniques, goal setting and strategies.	Median, 46 (IQR, 19–63) of 82 (77–90) sessions offered. Absent from program for 30 days or more: 1–6 months, (39/144, 27%); 7–18 months, (75/144, 52%).	Standard care, including education and health classes unrelated to weight	Mean between‐group weight difference: −3.2 kg (*P* = 0.002). 5% weight decline: Intervention, (55/144, 38%); control, (34/147, 23%) (*P* = 0.009).
Detke 2014^32^ (USA, Russia, Poland, Germany; RCT)	102 (50) 15.9 (1.5)	101 (47) 15.7 (1.5)	SCH, BPD, other^‡^	Multiple 52 weeks Individual (children, carers) Trained site personnel	Counselling sessions (education, problem solving, motivation); components: food, physical activity logs; healthy food diet; pedometers; review, goal setting; behaviour modification strategies.	No information	Baseline 15‐minute information session on healthy eating and exercise	Mean change in BMI (52 weeks): No differences between groups. 15% or greater weight gain: Intervention, (31/101, 31%); control, (41/102, 40%; *P* = 0.19).
Erickson 2016^33^ (USA; RCT)	48 (6) 49.6 (9.1)	60 (6) 49.7 (6.9)	SCH, BPD, other^‡^	Diet/nutrition 52 weeks Mixed Dietitian/ health instructor	Eight weekly education classes (with monthly boosters): dietary monitoring; recommendations for calorie deficit (food, physical activity logs); individual coaching on nutrition, lifestyle; optional group physical activity classes.	Mean: 13.7 of 19 sessions. Completed program: 25/60 (42%).	Standard care	Predicted mean weight change: Intervention, –4.6 kg; control, +0.6 kg.
Erickson 2017^34^ (USA; RCT)	42 (10) 50.4 (9.0)	62 (10) 51.9 (9.3)	SCH, BPD, other^‡^	Diet/nutrition 52 weeks Mixed Dietitian	Eight weekly classes (with monthly boosters): effects of medications; stress management; motivation, goal setting; diet, nutrition (mindful eating, portion sizes, calories, food groups, variety); physical activity. Food, physical activity journals.	Completed initial program: 8 weeks, 53/62 (86%); 12 months, 33/62 (53%). Completed journals: 57/62 (92%).	Sessions with study team, including anthropometric measurements and health‐related education.	Waist circumference, mean change: intervention, –1.04 cm; control, –0.25 cm (*P* < 0.001) Body fat, mean change: Intervention, –0.4 percentage points; control, +0.2 percentage points (*P* = 0.038).
Errichetti 2020^35^ (USA; RCT)	167 (93) 40.7 (13.4)	249 (137) 41.0 (12.5)	SCH, BPD, MDD, other^‡^	Add on service 52 weeks Individual Dietitian	Referral process with care coordinator based on individual needs and comorbid conditions.	Retention rates: intervention 154/249 (62%); control 115/167 (69%).	Standard care	Systolic blood pressure, HbA_1c_: Improvement greater for intervention: mean differences, –3.86 mmHg (SD, 1.89), *P* = 0.04; –0.36% (SD, 0.11), *P* = 0.001.
Frank 2015^36^ (USA; RCT)	61 (NR) 41.4 (9.7)	61 (NR) 41.8 (9.5)	BPD	Multiple 104 weeks Mixed Lifestyle coach	Fifteen individual sessions (with monthly group sessions): 3 × psychoeducation (risks, lifestyle); 4 × healthy sleep (relationship with mood, habits, lifestyle, social rhythmicity, relapse prevention); 4 × weight loss/nutrition (healthy eating; calories, physical activity, alcohol, relapse prevention); 4 × weight loss/physical activity (regular physical activity, variety; motivation, relapse prevention); 2 × optional smoking cessation.	At least one study visit: 58/61 (95%).	Standard care with medical monitoring	Between‐group difference in mean BMI reduction: Greater for intervention (0.51; 95% CI, 0.14–0.91).
Goldberg 2013^37^ (USA; RCT)	56 (8) 53.5 (8.1)	53 (13) 50.5 (9.9)	SCH, BPD, MDD, other^‡^	Multiple 26 weeks Mixed Research assistant	Weekly individual (first month) and group sessions (2‐4 months, weekly; 5‐6 months, fortnightly): healthy eating (history, planning); motivation, engagement; physical activity for weight loss; goal setting; successes, challenges; review of concepts, strategies, skills.	Completed 6‐month assessment: 30/53 (57%). Completed all individual sessions: 37/53 (70%). Competed 12 or more group sessions: 18/53 (34%); fewer than 4: 19/53 (36%).	Basic information on diet and exercise and monthly weight check	Reduction in weight > 5%: Overall: 7/109 (6%); no difference between groups. No difference between participants who completed at least eight sessions and those who completed fewer sessions.
Green 2015^38^ (USA; RCT)	96 (69) 48.3 (9.7)	104 (75) 46.2 (11.4)	SCH, BPD, other^‡^	Multiple 52 weeks Mixed Facilitators (mental health counsellor, nutrition interventionist, group leader)	Weekly 2 h group meetings (incl. 20 min physical activity) for 6 months; 6‐monthly group sessions; monthly phone calls. Individual records (dietary intake, daily physical activity, nightly sleep) with goals for daily moderate physical activity, increase fruit, vegetable, low‐fat dairy intake, improve sleep. Personalised plans, group work to improve skills, overcome barriers.	Attendance during first 6 months: mean, 14.5 (SD, 7.2) of 24 sessions (60%).	Standard care	Mean weight change: 6 months: intervention *v* control, –4.4 kg (95% CI, –6.96 kg to –1.78kg) 12 months: –2.6 kg (95% CI, –5.14 kg to –0.07 kg). During maintenance (6–12 months): no difference.
Holt 2019^39^ (UK; RCT)	205 (110) 40.1 (11.5)	207 (92) 40.0 (11.3)	SCH	Multiple 52 weeks Mixed Trained facilitator	Four weekly 2.5 h group sessions; fortnightly individual contact (mostly phone) 10 min; 2.5 h group booster sessions at 4, 7, 10 months. Topics: weight control (healthy drinks, snacks, calories, portions, food choices); physical activity, sedentary behaviour. Tools: water bottle, pedometer, cookbook/food scale; weight scale/tape measure.	Attended three or more sessions and at least one booster session: 111 (54%); 47 (23%) attended all sessions, 36 (17%) no sessions.	Basic printed advice on lifestyle and weight‐related risk factors	Mean between‐group difference in weight loss (12 months): No difference: 0.0 kg (95% CI, −1.6 to 1.7).
Iglesias‐Garcia 2010^40^ (Spain; RCT)	7 (3) 39.9 (11.3)^†^	8 (3) 39.9 (11.3)^†^	SCH	Multiple 12 weeks Group Nurse	12 weekly 1 h group sessions for 3 months: education on nutrition, exercise, healthy habits, self‐esteem; group discussions.	One participant withdrew consent.	Weekly anthropometric assessments	Waist circumference: Similar decline in both groups. BMI, weight: No difference within or between groups.
Jelalian 2019^41^ (USA; RCT)	9 (7) 14.4 (1.7)	24 (17) 15.3 (1.5)	MDD	CBT and lifestyle 24 weeks Mixed Multiple‐ team	Weekly 60 min sessions (weeks 1– 12), fortnightly sessions (weeks 13–24); weekly 60 min physical activity sessions. CBT protocol: problem solving, cognitive restructuring, affect regulation, behavioural activation (additional modules as needed). Diet component: body image; food craving/choices; individualised recommendations.	Mean, nine physical activity sessions and two diet/nutrition sessions	CBT protocol only	Expected change in BMI over 12 months: Intervention, +0.6 kg/m^2^; control, +2.1 kg/m^2^.
Kilbourne 2013^24^ (USA; RCT)	59 (10) 52.4 (9.2)	57 (10) 53.1 (10.6)	BPD	Multiple 52 weeks Group Health specialist	Four weekly 90–120 min sessions: blood pressure; personal and behavioural risk factors for CVD; personal goals; coping strategies; engagement and communication. Two sessions specifically on behavioural changes: avoiding over‐eating; using physical activity for stress reduction. Follow up monthly for 12 months.	Completed three or more weekly sessions: 39/57 (68%); mean, 4.6 (SD, 3.6) follow‐up sessions.	Standard enhanced care, including regular discussion of wellness	Blood pressure: Greater declines in intervention group: systolic: beta = –3.1, *P* = 0.04; diastolic: beta = –2.1, *P* = 0.04.
Looijmans 2019^43^ (Netherlands; CCT)	104 (50) 48.6 (10.2)	140 (74) 44.3 (10.9)	Psychosis, other^‡^	Multiple 52 weeks Mixed Nurse	Screening phase: appraisal of lifestyle behaviour (Traffic Light Method); development of a plan with SMART goals (diet/physical activity based on guidelines). Follow‐up phase: fortnightly 15 min visits for 6 months for assessment of progress. After 6 months: screening/ adjustment goals/plans. Overall: 26 visits (23 reports) in 12 months.	Completed lifestyle screening, developed plans, goals: 108/140 (77%). No further reports for 13; 60 with median 4 reports, 35 with median 14 reports.	Standard care inclusive of lifestyle guidance if requested by participant	Waist circumference change: No between‐group differences in change at 6 months (−0.15 cm; (95% CI,−2.49 to –2.19) or 12 months (−1.03 cm; 95% CI, −3.42 to –1.35).
Lovell 2014^44^ (UK; RCT)	51 (21) 25.9 (6.0)	54 (21) 25.6 (5.5)	SCH, other^‡^	Multiple 52 weeks Mixed Trained recovery worker	Seven sessions over 6 months; booster session at 9 or 10 months: psychoeducation for motivation; facilitation of changes in physical activity, diet with goals/reviews. Cooking groups; booklet, website (plans, goals, recipes).	At least one session: 54/56 (96%); 42/56 (78%) completed 6–8 sessions.	Standard early intervention care. If part of the care plan, control participants received some physical health support from their case manager	Change in BMI: No significant differences.
Magni 2017^45^ (Italy; nRCT)	26 (11) 41.8 (10.1)	59 (32) 43.1 (9.0)	SCH	Multiple 16 weeks Group Multiple (team)	32 twice weekly 1 h sessions: nutrition psychoeducation (information and recommendations for calorie‐restricted diet); cognitive bias regarding food habits; self‐observation of eating behaviour and emotional eating; alternative behaviours. Mediterranean diet‐based diet plan, physical activity plan.	No information.	Standard care, including information on food and nutrition	Mean change in BMI: Intervention, –1.9% (from 32.6 to 32.0); control, +0.6% (from 35.0 to 35.2; *P* = 0.021).
Masa‐Font 2015^46^ (Spain; RCT)	163 (74) 47.1 (9.9)	169 (76) 46.3 (8.9)	SCH, BPD	Multiple 12 weeks Group Nurse	24 twice weekly physical activity sessions for 3 months: 8 × 40 min sessions on intensity, safety of physical activity; 16 × 60 min walking sessions. 6 × 20 min twice weekly healthy dietary habits sessions (Mediterranean diet, review of food consumption).	Intervention participation at 3 months: 142/169 (84%). Attended 60% of sessions: 83/169 (49%); attended no sessions: 21 (6%).	Standard care	Mean change in BMI: Intervention, +0.04 kg/m^2^ (95% CI, –0.15 to +0.22); control, –0.23 kg/m^2^ (95% CI, –0.39 to –0.07).
Methapatara 2011^47^ (Thailand; RCT)	32 (14) 37.6 (10.8)	32 (9) 43.2 (9.3)	SCH	Multiple 12 weeks Mixed Researcher	Five 1 h sessions: motivational interviewing; adequate physical activity; education on nutrition, physical activity; SMART goal setting; supervised walking; feedback, coping strategies.	All participants completed all sessions.	Standard care, including informational leaflet on healthy lifestyle	Mean between group difference in weight loss: Intervention *v* control: 2.2 kg (95% CI, 0.29–4.12).
Osborn 2018^48^ (UK; CCT)	172 (84) 51.0 (10.0)	155 (88) 51.0 (10.0)	SCH, BPD	Multiple 52 weeks Individual Nurse/ health care assistant	Weekly or fortnightly appointments for 6 months to set goals/health care plans (medication adherence, improving diet, increase physical activity, reduce alcohol, quit smoking)	Attended six or more appointments: 72/155 (46%); 36 (23%) attended 2–5; 15 (10%) one appointment and 32 (21%) none.	Standard care	Mean serum triglycerides (12 months): Intervention, 5.4 mmol/L (SD, 1.1); control, 5.5 mmol/L (SD, 1.1); estimated mean difference, 0.03 mmol/L (95% CI, –0.22 to 0.29).
Sugawara 2018^49^ (Japan; RCT)	85 (26) 44.0 (10.3)	Group 1: 67 (36) 47.6 (9.6) Group 2: 61 (29) 46.6 (10.9)	SCH	Diet/nutrition 52 weeks Individual Dietitian/ doctor	Two intervention groups: 1. Weight loss advice from psychiatrist (record book, target body weight). 2. Twelve monthly sessions with dietitian, four phases, each 3 × 30–40 min sessions: balanced diet; food choices; food requirements; revision and discussion of food records.	Completed intervention: group 1, 67/93; group 2, 61/87.	Standard care	Mean weight change: Greater decline for group 2 (–3.2 kg; SD, 4.5) than group 1 (–0.4 kg; SD, 3.9) and control (+0.5 kg; SD, 5.1).
Sylvia 2019^50^ (UK; RCT)	19 (12) 44.3 (11.9)	19 (13) 39.7 (12.5)	BPD	Multiple 20 weeks Individual Senior students	Three modules: nutrition (sessions 1–6: education, skills to improve choices, portion control); exercise (sessions 7–12: moderate physical activity goals); wellness (sessions 13–18: healthy decisions, problem solving).	Attended sessions: 13/19 (67%)	Standard care	Mean weight change: No significant change in either group.
Verhaeghe 2013^15^ (Belgium; CCT)	83 (28) 46.6 (11.9)	201 (82) 46.2 (12.5)	SCH, other^‡^	Multiple 10 weeks Mixed Nurses	Weekly group sessions: physical activity, healthy eating, problem solving; written exercises /plans. Weekly 30 min group walking. 10 min individual sessions to follow up group activities, discuss challenges, arrange next session.	Attended 8 of 10 sessions: 103/201 (51%).	Standard care	Mean weight change: Intervention, −0.35 kg; control, +0.22 kg; *P* = 0.04), Mean BMI change: Intervention, −0.12 kg/m^2^; control, +0.08kg/m^2^; *P* = 0.04), Mean waist circumference change: Intervention, −0.29 cm; control, +0.55 cm; *P* < 0.01) Mean percentage body fat change: Intervention, −0.99 percentage points; control; −0.12 percentage points; *P* < 0.01). All differences, except for body fat, lost by 6‐month follow‐up.

BMI = body mass index; BPD = bipolar disorder; CBT = cognitive behavioural therapy; CCT = cluster controlled trial; CI = confidence interval; MDD = major depressive disorder; NR = not reported; nRCT = non‐randomised controlled trial; RCT = randomised controlled trial; SCH = schizophrenia or schizoaffective disorder; SD = standard deviation.

* Type (diet/nutrition only or multiple component only intervention).

^†^ Age data were reported for both samples combined; ie, age data by group unavailable.

^‡^ Includes mood disorders, post‐traumatic stress disorder, other psychotic disorders.

Ten studies enrolled people with schizophrenia and related psychoses,^27^, ^29^, ^38^, ^39^, ^40^, ^44^, ^45^, ^47^, ^49^, ^51^ seven studies people with schizophrenia, related psychosis, or bipolar disorder,^28^, ^32^, ^33^, ^34^, ^43^, ^46^, ^48^ three studies people with bipolar affective disorder,^36^, ^42^, ^50^ one study people with depression,^41^ and four studies enrolled people with any serious mental illness.^30^, ^31^, ^35^, ^37^ Study sample sizes (intervention and control arms total) ranged from 15^40^ to 416.^35^

All studies included both men and women. Twenty studies enrolled people living in the community,^27^, ^28^, ^30^, ^31^, ^33^, ^34^, ^35^, ^36^, ^37^, ^38^, ^39^, ^40^, ^41^, ^42^, ^44^, ^46^, ^48^, ^49^, ^50^, ^51^ four studies both psychiatric hospital inpatients and people dwelling in the community,^32^, ^43^, ^45^, ^47^ and one study inpatients only.^29^ Twenty‐one studies included participants whose mean age was greater than 30 years.^27^, ^28^, ^29^, ^31^, ^33^, ^34^, ^35^, ^36^, ^37^, ^38^, ^39^, ^40^, ^42^, ^43^, ^45^, ^46^, ^47^, ^48^, ^49^, ^50^, ^51^ Three studies delivered nutrition interventions only,^33^, ^34^, ^49^ 22 studies delivered nutrition interventions as part of a broader lifestyle intervention.^27^, ^28^, ^29^, ^30^, ^31^, ^32^, ^35^, ^36^, ^37^, ^38^, ^39^, ^40^, ^41^, ^42^, ^43^, ^44^, ^45^, ^46^, ^47^, ^48^, ^50^, ^51^ Fourteen interventions were implemented as combinations of group and individual sessions,^28^, ^30^, ^31^, ^33^, ^34^, ^36^, ^37^, ^38^, ^39^, ^41^, ^43^, ^44^, ^47^, ^51^ six only in group sessions,^27^, ^29^, ^40^, ^42^, ^45^, ^46^ and five as individual‐level interventions only.^32^, ^35^, ^48^, ^49^, ^50^ In eight studies, specialist clinicians (dietitian or equivalent) delivered the nutrition intervention;^27^, ^28^, ^29^, ^30^, ^33^, ^34^, ^35^, ^49^ the other studies used non‐specialist clinicians or other workers for this purpose. The duration of nineteen studies exceeded twelve weeks.^28^, ^29^, ^31^, ^32^, ^33^, ^34^, ^35^, ^36^, ^37^, ^38^, ^39^, ^41^, ^42^, ^43^, ^44^, ^45^, ^48^, ^49^, ^50^

### Risk of bias and certainty of evidence

Eight studies were deemed to be at low or some risk of bias,^37^, ^38^, ^39^, ^41^, ^44^, ^46^, ^47^, ^50^ seventeen were deemed to be at high risk.^27^, ^28^, ^29^, ^30^, ^31^, ^32^, ^33^, ^34^, ^35^, ^36^, ^40^, ^42^, ^43^, ^45^, ^48^, ^49^, ^51^ Risk was generally linked with low adherence by participants to intervention measures (Supporting Information, figures 1–5). Certainty of evidence was low for all outcome measures except LDL‐cholesterol (very low certainty) (Supporting Information, table 7).

### Strength and consistency of intervention effect on primary outcomes

Eight of seventeen intervention arms found statistically significant intervention effects on weight,^28^, ^30^, ^31^, ^34^, ^38^, ^47^, ^49^, ^51^ ten of 24 intervention arms on BMI,^30^, ^31^, ^33^, ^34^, ^36^, ^38^, ^45^, ^47^, ^49^, ^51^ and seven of seventeen on waist circumference.^29^, ^30^, ^31^, ^33^, ^47^, ^49^, ^51^ Two of the eight studies with low or some risk of bias found statistically significant effects of interventions on weight, BMI, or waist circumference.^38^, ^47^

### Longer term follow‐up

Three studies that undertook longer term follow‐up after the intervention each found statistically significant intervention effects on a primary outcome.^27^, ^29^, ^51^ In one study, the difference between intervention and control group was not statistically significant at the end of the 3‐month intervention but was significant at the 6‐month follow‐up.^27^ A second study found a significant effect on waist circumference at the end of the 24‐week intervention and also at 48 weeks; further, blood glucose levels were lower in the intervention group at 48 weeks, but there was no effect on weight at either time point.^29^ A third study found significant effects on weight and BMI, waist circumference, and body fat at the end of the 10‐week intervention, but only in body fat at 6‐month follow‐up.^51^

### Pooled effect of nutrition interventions on metabolic syndrome risk factors

Meta‐analysis did not identify statistically significant intervention effects on weight (eleven studies; 810 intervention, 682 control participants; SMD, –0.11; 95% CI, –0.29 to 0.06), BMI (16 studies; 1605 intervention, 1258 control participants; SMD, 0.01; 95% CI, –0.32 to 0.33), or waist circumference (12 studies; 1197 intervention, 1044 control participants; SMD, –0.02; 95% CI, –0.17 to 0.13) (Box 3).

Box 3 **Forest plots for trials that assessed the impact of diet or nutrition‐based interventions on primary outcomes (inverse variance, random)**

**Figure:**
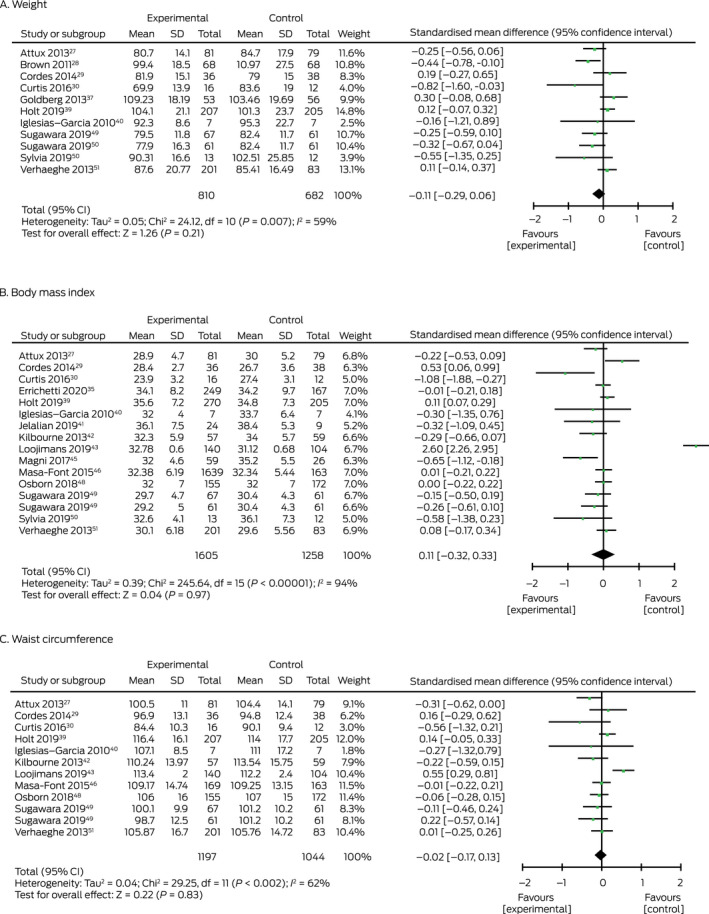
df = degrees of freedom; SD = standard deviation. ◆

Subgroup analysis found a small effect size on weight for interventions delivered by dietitians (five studies; 262 intervention, 258 control participants; SMD, –0.28; 95% CI, –0.51 to –0.04) (Supporting Information, figure 6) and interventions based on individual sessions only (three studies; 141 intervention, 134 control participants; SMD, –0.30; 95% CI, –0.54 to –0.06) (Supporting Information, figure 7). Despite some asymmetry in the funnel plot, publication bias was not significant (Supporting Information, figure 8).

Meta‐analysis did not identify statistically significant effects of interventions on blood pressure, serum lipid, or blood glucose levels (Box 4). For the three interventions delivered by dietitians (391 intervention, 307 control participants; *I* ^2^ = 0%),^27^, ^35^, ^49^ subgroup analysis identified small effects on systolic (SMD, –0.18; 95% CI, –0.34 to –0.03) and diastolic blood pressure (SMD, –0.18, 95% CI, –0.33 to –0.02) (Supporting Information, figure 9).

Box 4 **Forest plots for trials that assessed the impact of diet or nutrition‐based interventions on secondary outcomes (inverse variance, random)**

**Figure:**
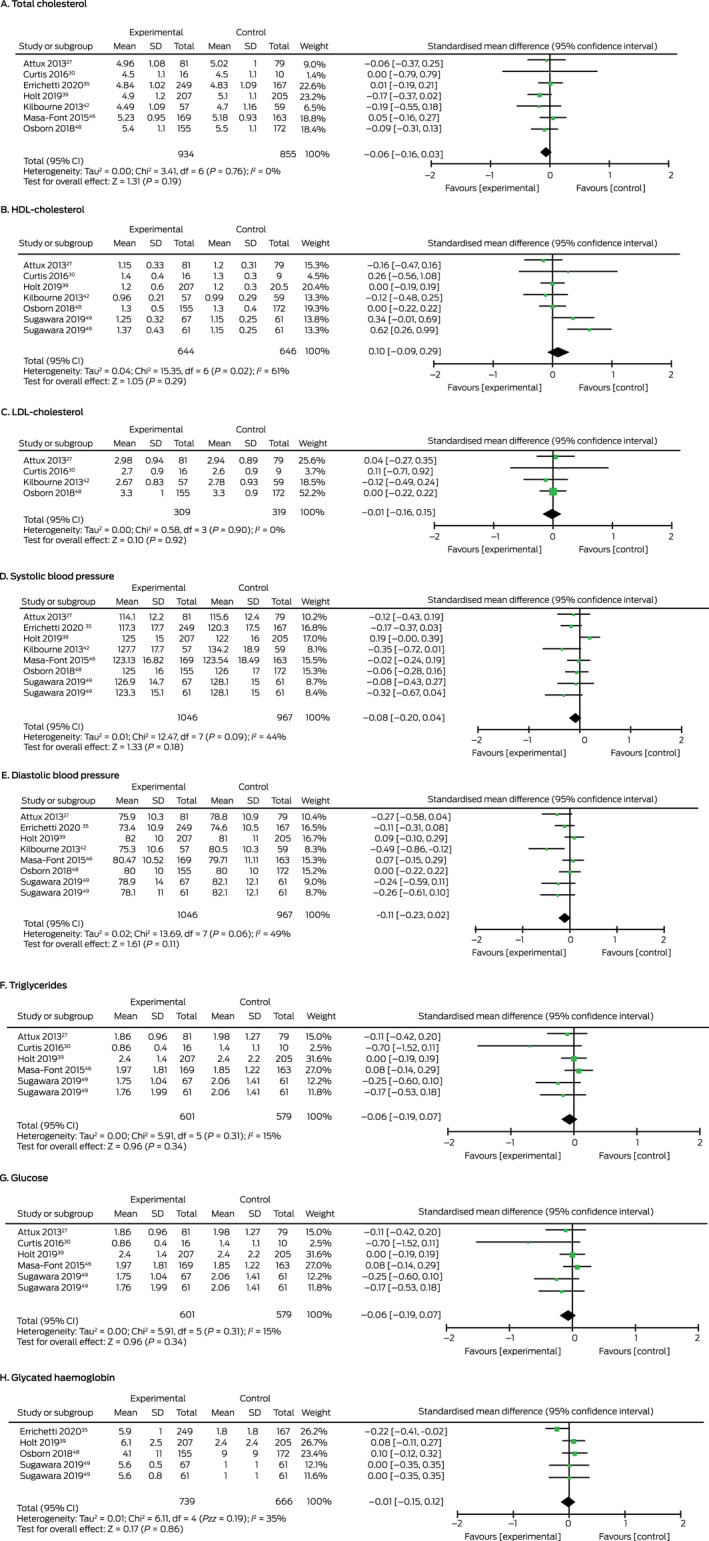
df = degrees of freedom; HDL = high‐density lipoprotein; LDL = low‐density lipoprotein; SD = standard deviation. ◆

## Discussion

Our review of 25 randomised and non‐randomised trials published during 2010–2021 found limited evidence that nutrition interventions improve metabolic syndrome risk factors in people with serious mental illness. However, such interventions may be more effective when delivered on an individual basis or by dietitians.

Individualised and dietitian‐delivered sessions may be effective because dietitians can assess and respond to the unique nutrition‐related challenges experienced by people living with serious mental illness. Moreover, dietitians can provide appropriate interventions that incorporate behaviour change techniques, specific goals, and self‐monitoring. Each of the individualised (completely individual‐based, or including both individual and group components) and dietitian‐delivered interventions we reviewed found at least one favourable effect on a metabolic syndrome risk factor. Our finding complements that of an earlier systematic review and meta‐analysis,^20^ which found that nutrition interventions delivered by dietitians or early in the course of antipsychotic medication use were the most effective.

The poorer physical health and reduced life expectancy of people living with serious mental illness in high income countries has been labelled a “scandal”,^52^ and it is a priority area for action for all governments in Australia.^53^ The 2019 *Lancet Psychiatry* commission report^13^ provided recommendations on multidisciplinary approaches to managing physical and mental multimorbidity, including six elements for effective lifestyle interventions:

- include both dietary modification and exercise;
- use behaviour change techniques, including specific and measurable goals, and self‐monitoring;
- be delivered by staff with professional qualifications in nutrition, dietetics, and exercise;
- offer supervised exercise sessions at least twice a week;
- familiarise mental health staff with the lifestyle intervention; and
- include peer support.

Several of these elements were missing from most interventions we reviewed, perhaps explaining why they were not effective. The 2020 Australian Mental Health Productivity Commission Inquiry Report^54^ stated that the mental health system needs to provide holistic and person‐centred care focused on the individual and their life circumstances to effectively improve the physical health of people with serious mental illness.

Such programs have high attrition and low adherence rates,^55^ but intervention acceptance, adherence, and retention could be enhanced by incorporating the elements outlined by the *Lancet Psychiatry* commission:^13^ embedding the intervention in mental health services to avoid disconnection from other health services, familiarising mental health staff with the intervention, integrating peer workers to help people navigate the health service and lifestyle intervention and to assist with health coaching and follow‐up, and providing affordable supervised exercise sessions. Further, our analysis indicates that nutrition interventions should include individualised components and be delivered by a nutrition professional (eg, a dietitian).

A recent scoping review^56^ of economic studies of dietary interventions for people with a variety of mental disorders included five cost‐effectiveness studies^48^, ^57^, ^58^, ^59^, ^60^ associated with five trials^31^, ^38^, ^39^, ^48^, ^51^ we included in our review. The findings and the strength of conclusions that could be derived from the scoping review were limited, but provided some preliminary information. For example, the cost analysis for the successful ACHIEVE trial of a behavioural weight loss intervention in the community found that it could be offered at a cost of $US65 to $US85 per person per month.^60^ If dietitian‐led and individualised interventions are effective for reducing metabolic syndrome risks, employing these interventions more widely could achieve net cost savings for healthcare systems. Without comprehensive economic evaluations, however, it is unclear whether the investment would meet accepted cost‐effectiveness/utility thresholds.

### Strengths and limitations of our review

Strengths included our rigorous search strategy; the identification of a reasonable number of relevant trials; the independent screening, data extraction, and study and outcome appraisal by several review authors; our assessments of risk of bias, GRADE, and study data strength and consistency; and our identification of elements associated with favourable intervention outcomes that could guide future trials and clinical practice.

However, as we restricted our review to studies published since 2010, our conclusions are limited to understanding the elements and effectiveness of more recent interventions. We excluded studies published in languages other than English, potentially biasing our findings. The small proportion of studies that could be pooled for meta‐analysis suggests that the estimated effect sizes may not properly reflect the findings of all publications included in our review. We therefore assessed the proportion of studies that reported significant intervention effects. Study quality was generally poor; most were found to be at high risk of bias, and risk was low for only one study.^44^ Study heterogeneity was marked with respect to the primary analyses, in part because of variation in study design and intervention delivery, and low study quality. Heterogeneity was much lower and not statistically significant in subgroup analyses of dietitian‐delivered interventions and individualised interventions.

## Conclusion

Our results provide only limited evidence for nutrition interventions improving metabolic syndrome risk factors in people with serious mental illness, but they could be effective when delivered on an individual basis or by dietitians. Further trials could explore this question, and also assess the cost‐effectiveness of such interventions.

## Open access

Open access publishing facilitated by University of New South Wales, as part of the Wiley – University of New South Wales agreement via the Council of Australian University Librarians.

## Competing interests

No relevant disclosures.

## Supporting information


Appendix S1.
