# Supplementary material for: Effectiveness of nutrition and dietary interventions for people with serious mental illness: systematic review and meta‐analysis
Source: Med J Aust. 2022 Oct 2;217(Suppl 7):S7–S21. doi: 10.5694/mja2.51680 (PMC9828433; doi:10.5694/mja2.51680)
Supplement: Supplementary file 1 — Appendix S1. [file MJA2-217-S7-s001.pdf]

MJA
The Medical Journal of Australia

Supporting Information

Supplementary methds and results

This appendix was part of the submitted manuscript and has been peer reviewed.
It is posted as supplied by the authors.

Appendix to: Rocks T, Teasdale SB, Fehily C, et al. Effectiveness of nutrition and dietary interventions for people with serious mental illness: systematic review and meta-analysis. *Med J Aust* 2022; doi: 10.5694/mja2.51680.

Table 1. Search strategy for MEDLINE Complete. Searched via EBSCOhost on 26 March 2021 and repeated on 6 September 2021

S#	Search string
S101	S100<br>Limiters - Date of Publication: 20100101-; English Language
S100	S98 NOT S99
S99	MH "Animals+" NOT MH "Humans"
S98	S15 AND S34 AND S69 AND S97
S97	S85 OR S96
S96	S86 OR S87 OR S88 OR S89 OR S90 OR S91 OR S92 OR S93 OR S94 OR S95
S95	MH "Blood Glucose"
S94	MH "Lipids+"
S93	MH "Blood Pressure"
S92	MH “Waist Circumference+”
S91	MH "Overweight+"
S90	MH “Weight Loss”
S89	MH “Body Weight”
S88	MH “Weight Gain”
S87	MH “Body Composition+”
S86	MH “Body Mass Index”
S85	S70 OR S71 OR S72 OR S73 OR S74 OR S75 OR S76 OR S77 OR S78 OR S79 OR S80 OR S81 OR S82 OR S83 OR S84
S84	TI “blood glucose” OR AB “blood glucose”
S83	TI lipid* OR AB lipid*
S82	TI “blood pressure*” OR AB “blood pressure*”
S81	TI "body fat" OR AB "body fat"
S80	TI adipos* OR AB adipos*
S79	TI "waist circumference*" OR AB "waist circumference*"
S78	TI "lost weight" OR AB "lost weight"
S77	TI "lose weight" OR AB "lose weight"
S76	TI "weight loss" OR AB "weight loss"
S75	TI "gain* weight" OR AB "gain* weight"
S74	TI "weight gain" OR AB "weight gain"
S73	TI BMI OR AB BMI

S72	TI "body mass index" OR AB "body mass index"
S71	TI "body composition" OR AB "body composition"
S70	TI "body weight" OR AB "body weight"
S69	S56 OR S68
S68	S57 OR S58 OR S59 OR S60 OR S61 OR S62 OR S63 OR S64 OR S65 OR S66 OR S67
S67	MH “Clinical Trials as Topic+”
S66	MH “Treatment Outcome”
S65	PT "validation study"
S64	PT "randomized controlled trial"
S63	PT "pragmatic clinical trial"
S62	PT "multicenter study"
S61	PT "evaluation study"
S60	PT "equivalence trial"
S59	PT "controlled clinical trial"
S58	PT "comparative study"
S57	PT "clinical trial"
S56	S35 OR S36 OR S37 OR S38 OR S39 OR S40 OR S41 OR S42 OR S43 OR S44 OR S45 OR S46 OR S47 OR S48 OR S49 OR S50 OR S51 OR S52 OR S53 OR S54 OR S55
S55	TI "follow up" OR AB "follow up"
S54	TI "as usual" OR AB "as usual"
S53	TI "standard care" OR AB "standard care"
S52	TI experiment* OR AB ex<br>periment*
S51	TI programmes OR AB programmes
S50	TI programme OR AB programme
S49	TI programs OR AB programs
S48	TI program OR AB program
S47	TI interventions OR AB interventions
S46	TI intervention OR AB intervention
S45	TI rct OR AB rct
S44	TI controlled OR AB controlled
S43	TI control OR AB control
S42	TI blinded OR AB blinded
S41	TI blinding OR AB blinding

S40	TI blind OR AB blind
S39	TI groups OR AB groups
S38	TI placebo OR AB placebo
S37	TI random* OR AB random*
S36	TI trial OR AB trial
S35	TI strateg* OR AB strateg*
S34	S28 OR S33
S33	S29 OR S30 OR S31 OR S32
S32	MW dh
	MH “Overweight+ REMOVE THIS SEARCH LINE AND ADJUST S# NUMBERING
S31	MH “Diet Therapy+“
S30	MH “Weight Reduction Programs+”
S29	MH “Diet+“
S28	S16 OR S17 OR S18 OR S19 OR S20 OR S21 OR S22 OR S23 OR S24 OR S25 OR S26 OR S27
S27	TI nutri* OR AB nutri*
S26	TI dieticians OR AB dieticians
S25	TI dietician OR AB dietician
S24	TI dietitians OR AB dietitians
S23	TI dietitian OR AB dietitian
S22	TI dieting OR AB dieting
S21	TI dietetics OR AB dietetics
S20	TI dietetic OR AB dietetic
S19	TI dieted OR AB dieted
S18	TI dietary OR AB dietary
S17	TI diets OR AB diets
S16	TI diet OR AB diet
S15	S10 OR S14
S14	S11 OR S12 OR S13
S13	MH "Schizophrenia Spectrum and Other Psychotic Disorders+"
S12	MH "Bipolar Disorder"
S11	MH "Depressive Disorder+"
S10	S1 OR S2 OR S3 OR S4 OR S5 OR S6 OR S7 OR S8 OR S9
S9	TI psychotic OR AB psychotic
S8	TI psychoses OR AB psychoses

S7	TI psychosis OR AB psychosis
S6	TI schizoaffective OR AB schizoaffective
S5	TI schizophreni* OR AB schizophreni*
S4	TI bipolar depression OR AB bipolar depression
S3	TI bipolar disorder* OR AB bipolar disorder*
S2	TI unipolar depression OR AB unipolar depression
S1	TI unipolar disorder* OR AB unipolar disorder*

Table 2. Search strategy for Embase. Searched via Embase.com on 26 March 2021 with and repeated on 6 September 2021

#	**Search string**
#76	#15 AND #34 AND #74 AND [english]/lim AND [2010-2021]/py
#75	#15 AND #34 AND #74
#74	#57 OR #73
#73	#58 OR #59 OR #60 OR #61 OR #62 OR #63 OR #64 OR #65 OR #66 OR #67 OR #68 OR #69 OR #70 OR #71 OR #72
#72	'quasi experimental study'/de
#71	'experimental study'/de
#70	'clinical trial (topic)'/de
#69	'treatment failure'/de
#68	'patient-reported outcome'/de
#67	'outcomes research'/de
#66	'outcome assessment'/de
#65	'minimal clinically important difference'/de
#64	'clinical outcome'/de
#63	'treatment outcome'/de
#62	'open study'/de
#61	'major clinical study'/de
#60	'intervention study'/de
#59	'community trial'/de
#58	'clinical trial'/exp
#57	#35 OR #36 OR #37 OR #38 OR #39 OR #40 OR #41 OR #42 OR #43 OR #44 OR #45 OR #46 OR #47 OR #48 OR #49 OR #50 OR #51 OR #52 OR #53 OR #54 OR #55 OR #56
#56	'follow up':ab,ti
#55	'as usual':ab,ti
#54	'standard care':ab,ti
#53	experiment*:ab,ti
#52	programmes:ab,ti
#51	programme:ab,ti
#50	programs:ab,ti
#49	program:ab,ti
#48	interventions:ab,ti
#47	intervention:ab,ti
#46	rct:ab,ti
#45	controlled:ab,ti
#44	control:ab,ti
#43	blinded:ab,ti
#42	blinding:ab,ti
#41	blind:ab,ti
#40	groups:ab,ti

#39	'control group*':ab,ti
#38	placebo:ab,ti
#37	random*:ab,ti
#36	trial:ab,ti
#35	strateg*:ab,ti
#34	#28 OR #33
#33	#29 OR #30 OR #31 OR #32
#32	'obesity'/exp
#31	'diet therapy'/exp
#30	'weight loss program'/de
#29	'diet'/exp
#28	#16 OR #17 OR #18 OR #19 OR #20 OR #21 OR #22 OR #23 OR #24 OR #25 OR #26 OR #27
#27	nutri*:ab,ti
#26	dieticians:ab,ti
#25	dietician:ab,ti
#24	dietitians:ab,ti
#23	dietitian:ab,ti
#22	dieting:ab,ti
#21	dietetics:ab,ti
#20	dietetic:ab,ti
#19	dieted:ab,ti
#18	dietary:ab,ti
#17	diets:ab,ti
#16	diet:ab,ti
#15	#10 OR #14
#14	#11 OR #12 OR #13
#13	'schizophrenia'/exp
#12	'bipolar disorder'/exp
#11	'major depression'/de
#10	#1 OR #2 OR #3 OR #4 OR #5 OR #6 OR #7 OR #8 OR #9
#9	psychotic:ab,ti
#8	psychoses:ab,ti
#7	psychosis:ab,ti
#6	schizoaffective:ab,ti
#5	schizophreni*:ab,ti
#4	bipolar AND depression:ab,ti
#3	bipolar AND disorder*:ab,ti
#2	unipolar AND depression:ab,ti
#1	unipolar AND disorder*:ab,ti

Table 3. Search strategy for CINAHL Complete. Searched via EBSCOhost on 26 March 2021 and repeated on 6 September 2021

S#	Search String
S105	S104<br><br>Limiters - Published Date: 20100101-; English Language
S104	S102 NOT S103
S103	MH "Animals+" NOT MH "Human"
S102	S26 AND S44 AND S72 AND S101
S101	S88 OR S100
S100	S89 OR S90 OR S91 OR S92 OR S93 OR S94 OR S95 OR S96 OR S97 OR S98 OR S99
S99	MH "Blood Glucose"
S98	MH "Lipids+"
S97	MH "Blood Pressure"
S96	MH "Waist Circumference"
S95	MH "Obesity, Morbid"
S94	MH “Obesity”
S93	MH "Weight Loss"
S92	MH "Body Weight"
S91	MH “Weight Gain”
S90	MH “Body Composition+”
S89	MH “Body Mass Index”
S88	S73 OR S74 OR S75 OR S76 OR S77 OR S78 OR S79 OR S80 OR S81 OR S82 OR S83 OR S84 OR S85 OR S86 OR S87
S87	TI “blood glucose” OR AB “blood glucose”
S86	TI lipid* OR AB lipid*
S85	TI “blood pressure*” OR AB “blood pressure*”
S84	TI "body fat" OR AB "body fat"
S83	TI adipos* OR AB adipos*
S82	TI "waist circumference*" OR AB "waist circumference*"
S81	TI "lost weight" OR AB "lost weight"
S80	TI "lose weight" OR AB "lose weight"
S79	TI "weight loss" OR AB "weight loss"
S78	TI "gain* weight" OR AB "gain* weight"
S77	TI "weight gain" OR AB "weight gain"
S76	TI BMI OR AB BMI
S75	TI "body mass index" OR AB "body mass index"
S74	TI "body composition" OR AB "body composition"
S73	TI "body weight" OR AB "body weight"
S72	S66 OR S71
S71	S67 OR S68 OR S69 OR S70
S70	(PT “randomized controlled trial”)

S69	(PT “clinical trial”)
S68	(MH "Experimental Studies+")
S67	(MH "Treatment Outcomes")
S66	S45 OR S46 OR S47 OR S48 OR S49 OR S50 OR S51 OR S52 OR S53 OR S54 OR S55 OR S56 OR S57 OR S58 OR S59 OR S60 OR S61 OR S62 OR S63 OR S64 OR S65
S65	TI "follow up" OR AB "follow up"
S64	TI "as usual" OR AB "as usual"
S63	TI "standard care" OR AB "standard care"
S62	TI experiment* OR AB experiment*
S61	TI programmes OR AB programmes
S60	TI programme OR AB programme
S59	TI programs OR AB programs
S58	TI program OR AB program
S57	TI interventions OR AB interventions
S56	TI intervention OR AB intervention
S55	TI rct OR AB rct
S54	TI controlled OR AB controlled
S53	TI control OR AB control
S52	TI blinded OR AB blinded
S51	TI blinding OR AB blinding
S50	TI blind OR AB blind
S49	TI groups OR AB groups
S48	TI placebo OR AB placebo
S47	TI random* OR AB random*
S46	TI trial OR AB trial
S45	TI strateg* OR AB strateg*
S44	S39 OR S43
S43	S40 OR S41 OR S42
S42	MW dh
S41	(MH "Diet Therapy+")
S40	(MH "Diet+")
S39	S27 OR S28 OR S29 OR S30 OR S31 OR S32 OR S33 OR S34 OR S35 OR S36 OR S37 OR S38
S38	TI nutri* OR AB nutri*
S37	TI dieticians OR AB dieticians
S36	TI dietician OR AB dietician
S35	TI dietitians OR AB dietitians
S34	TI dietitian OR AB dietitian
S33	TI dieting OR AB dieting
S32	TI dietetics OR AB dietetics
S31	TI dietetic OR AB dietetic
S30	TI dieted OR AB dieted

S29	TI dietary OR AB dietary
S28	TI diets OR AB diets
S27	TI diet OR AB diet
S26	S10 OR S25
S25	S11 OR S12 OR S13 OR S14 OR S15 OR S16 OR S17 OR S18 OR S19 OR S20 OR S21 OR S22 OR S23 OR S24
S24	(MH "Organic Mental Disorders, Psychotic+")
S23	(MH "Delusions+")
S22	(MH "Postpartum Psychosis")
S21	(MH "Paranoid Disorders")
S20	(MH "Schizoaffective Disorder")
S19	(MH "Psychoses, Substance-Induced+")
S18	(MH "Capgras Syndrome")
S17	(MH "Psychotic Disorders")
S16	(MH "Affective Disorders, Psychotic")
S15	(MH "Schizophrenia+")
S14	(MH "Bipolar Disorder+")
S13	(MH "Premenstrual Dysphoric Disorder")
S12	(MH "Seasonal Affective Disorder")
S11	(MH "Depression+")
S10	S1 OR S2 OR S3 OR S4 OR S5 OR S6 OR S7 OR S8 OR S9
S9	TI psychotic OR AB psychotic
S8	TI psychoses OR AB psychoses
S7	TI psychosis OR AB psychosis
S6	TI schizoaffective OR AB schizoaffective
S5	TI schizophreni* OR AB schizophreni*
S4	TI bipolar depression OR AB bipolar depression
S3	TI bipolar disorder* OR AB bipolar disorder*
S2	TI unipolar depression OR AB unipolar depression
S1	TI unipolar disorder* OR AB unipolar disorder*

Table 4. Search strategy for APA PsycInfo. Searched via EBSCOhost on 26 March 2021 and repeated on 6 September 2021

S#	Search string
S76	S75<br>Limiters - Published Date: 20100101-; Language: English
S75	S73 NOT S74
S74	DE "Animals" OR DE "Animal Limb" OR DE "Animal Offspring" OR DE "Female Animals" OR DE "Infants (Animal)" OR DE "Invertebrates" OR DE "Male Animals" OR DE "Pets" OR DE "Service Animals" OR DE "Species Differences" OR DE "Vertebrates" OR DE "Invertebrates" OR DE "Arthropoda" OR DE "Echinodermata" OR DE "Mollusca" OR DE "Worms" OR DE "Vertebrates" OR DE "Amphibia" OR DE "Birds" OR DE "Fishes" OR DE "Mammals" OR DE "Pigs" OR DE "Reptiles"
S73	S15 AND S30 AND S54 AND S72
S72	S70 OR S71
S71	DE "Weight Control" OR DE "Weight Gain" OR DE "Weight Loss" OR DE "Body Mass Index" OR DE "Body Weight" OR DE "Birth Weight" OR DE "Overweight" OR DE "Underweight" OR DE "Weight Control" OR DE "Weight Gain" OR DE "Weight Loss" OR DE "Obesity" OR DE "Overweight" OR DE "Obesity" OR DE "Fatty Acids" OR DE "Lipids" OR DE "Fatty Acids" OR DE "Gangliosides" OR DE "Lipopolysaccharide" OR DE "Lipoproteins" OR DE "Blood Pressure" OR DE "Blood Sugar"
S70	S55 OR S56 OR S57 OR S58 OR S59 OR S60 OR S61 OR S62 OR S63 OR S64 OR S65 OR S66 OR S67 OR S68 OR S69
S69	TI “blood glucose” OR AB “blood glucose”
S68	TI lipid* OR AB lipid*
S67	TI “blood pressure*” OR AB “blood pressure*”
S66	TI "body fat" OR AB "body fat"
S65	TI adipos* OR AB adipos*
S64	TI "waist circumference*" OR AB "waist circumference*"
S63	TI "lost weight" OR AB "lost weight"
S62	TI "lose weight" OR AB "lose weight"
S61	TI "weight loss" OR AB "weight loss"
S60	TI "gain* weight" OR AB "gain* weight"
S59	TI "weight gain" OR AB "weight gain"
S58	TI BMI OR AB BMI
S57	TI "body mass index" OR AB "body mass index"
S56	TI "body composition" OR AB "body composition"
S55	TI "body weight" OR AB "body weight"
S54	S52 OR S53
S53	DE "Causal Analysis" OR DE "Randomized Clinical Trials" OR DE "Evidence Based Practice" OR DE "Placebo" OR DE "Random Sampling" OR DE "Treatment Effectiveness Evaluation" OR DE "Experimental Design" OR DE "Between Groups Design" OR DE "Clinical Trials" OR DE "Followup Studies" OR DE "Repeated Measures" OR DE "Single-Case Experimental Design" OR DE "Experiment Controls" OR DE "Experimental Methods" OR DE "Quasi Experimental Methods" OR DE "Randomized Controlled Trials"
S52	S31 OR S32 OR S33 OR S34 OR S35 OR S36 OR S37 OR S38 OR S39 OR S40 OR S41 OR S42 OR S43 OR S44 OR S45 OR S46 OR S47 OR S48 OR S49 OR S50 OR S51
S51	TI "follow up" OR AB "follow up"

S50	TI "as usual" OR AB "as usual"
S49	TI "standard care" OR AB "standard care"
S48	TI experiment* OR AB experiment*
S47	TI programmes OR AB programmes
S46	TI programme OR AB programme
S45	TI programs OR AB programs
S44	TI program OR AB program
S43	TI interventions OR AB interventions
S42	TI intervention OR AB intervention
S41	TI rct OR AB rct
S40	TI controlled OR AB controlled
S39	TI control OR AB control
S38	TI blinded OR AB blinded
S37	TI blinding OR AB blinding
S36	TI blind OR AB blind
S35	TI groups OR AB groups
S34	TI placebo OR AB placebo
S33	TI random* OR AB random*
S32	TI trial OR AB trial
S31	TI strateg* OR AB strateg*
S30	S28 OR S29
S29	DE "Diets" OR DE "Dietary Restraint" OR DE "Drinking Behavior" OR DE "Alcohol Drinking Patterns" OR DE "Water Intake" OR DE "Eating Behavior" OR DE "Binge Eating" OR DE "Chewing" OR DE "Dietary Restraint" OR DE "Food Refusal" OR DE "Fast Food" OR DE "Food" OR DE "Food Additives" OR DE "Food Insecurity" OR DE "Food Preparation" OR DE "Food Safety" OR DE "Food Addiction" OR DE "Food Allergies" OR DE "Food Deprivation" OR DE "Food Preferences" OR DE "Food Intake"
S28	S16 OR S17 OR S18 OR S19 OR S20 OR S21 OR S22 OR S23 OR S24 OR S25 OR S26 OR S27
S27	TI nutri* OR AB nutri*
S26	TI dieticians OR AB dieticians
S25	TI dietician OR AB dietician
S24	TI dietitians OR AB dietitians
S23	TI dietitian OR AB dietitian
S22	TI dieting OR AB dieting
S21	TI dietetics OR AB dietetics
S20	TI dietetic OR AB dietetic
S19	TI dieted OR AB dieted
S18	TI dietary OR AB dietary
S17	TI diets OR AB diets
S16	TI diet OR AB diet
S15	S10 OR S14
S14	S11 OR S12 OR S13

S13	DE "Psychosis" OR DE "Acute Psychosis" OR DE "Affective Psychosis" OR DE "Alcoholic Psychosis" OR DE "Capgras Syndrome" OR DE "Childhood Psychosis" OR DE "Chronic Psychosis" OR DE "Experimental Psychosis" OR DE "Hallucinosis" OR DE "Paranoia (Psychosis)" OR DE "Postpartum Psychosis" OR DE "Reactive Psychosis" OR DE "Schizophrenia" OR DE "Senile Psychosis" OR DE "Toxic Psychoses" OR DE "Acute Psychosis" OR DE "Acute Schizophrenia" OR DE "Alcoholic Psychosis" OR DE "Alcoholic Hallucinosis" OR DE "Childhood Psychosis" OR DE "Childhood Schizophrenia" OR DE "Hallucinosis" OR DE "Alcoholic Hallucinosis" OR DE "Paranoia (Psychosis)" OR DE "Folie A Deux" OR DE "Schizophrenia" OR DE "Acute Schizophrenia" OR DE "Catatonic Schizophrenia" OR DE "Childhood Schizophrenia" OR DE "Paranoid Schizophrenia" OR DE "Process Schizophrenia" OR DE "Schizoaffective Disorder" OR DE "Schizophrenia (Disorganized Type)" OR DE "Schizophreniform Disorder" OR DE "Undifferentiated Schizophrenia"
S12	DE "Bipolar Disorder" OR DE "Bipolar I Disorder" OR DE "Bipolar II Disorder" OR DE "Cyclothymic Disorder" OR DE "Mania" OR DE "Mania" OR DE "Hypomania"
S11	((DE "Major Depression" OR DE "Anaclitic Depression" OR DE "Dysthymic Disorder" OR DE "Endogenous Depression" OR DE "Late Life Depression" OR DE "Postpartum Depression" OR DE "Reactive Depression" OR DE "Recurrent Depression" OR DE "Treatment Resistant Depression") OR (DE "Seasonal Affective Disorder")) OR (DE "Premenstrual Dysphoric Disorder")
S10	S1 OR S2 OR S3 OR S4 OR S5 OR S6 OR S7 OR S8 OR S9
S9	TI psychotic OR AB psychotic
S8	TI psychoses OR AB psychoses
S7	TI psychosis OR AB psychosis
S6	TI schizoaffective OR AB schizoaffective
S5	TI schizophreni* OR AB schizophreni*
S4	TI bipolar depression OR AB bipolar depression
S3	TI bipolar disorder* OR AB bipolar disorder*
S2	TI unipolar depression OR AB unipolar depression
S1	TI unipolar disorder* OR AB unipolar disorder*

Table 5. Search strategy for Cochrane CENTRAL Register of Controlled Trials (CENTRAL). Searched via the Cochrane Library on 26 March 2021 (2021, Issue 3) and repeated on 6 September 2021 (2021, Issue 9)

#	Search string
#1	(unipolar disorder*):ti OR (unipolar disorder*):ab
#2	(unipolar depression):ti OR (unipolar depression):ab
#3	(bipolar disorder*)i OR (bipolar disorder*):ab
#4	(bipolar depression):ti OR (bipolar depression):ab
#5	(schizophreni*):ti OR (schizophreni*):ab
#6	(schizoaffective):ti OR (schizoaffective):ab
#7	(psychosis):ti OR (psychosis):ab
#8	(psychoses):ti OR (psychoses):ab
#9	(psychotic):ti OR (psychotic):ab
#10	{OR #1-#9}
#11	MeSH descriptor: [Depressive Disorder] explode all trees
#12	MeSH descriptor: [Bipolar Disorder] this term only
#13	MeSH descriptor: [Schizophrenia Spectrum and Other Psychotic Disorders] explode all trees
#14	{OR #11-#13}
#15	#10 OR #14
#16	(diet):ti OR (diet):ab
#17	(diets):ti OR (diets):ab
#18	(dietary):ti OR (dietary):ab
#19	(dieted):ti OR (dieted):ab
#20	(dietetic):ti OR (dietetic):ab
#21	(dietetics):ti OR (dietetics):ab
#22	(dieting):ti OR (dieting):ab
#23	(dietitian):ti OR (dietitian):ab
#24	(dietitians):ti OR (dietitians):ab
#25	(dietician):ti OR (dietician):ab
#26	(dieticians):ti OR (dieticians):ab
#27	(nutri*):ti OR (nutri*):ab
#28	{OR #16-#27}
#29	MeSH descriptor: [Diet] explode all trees
#30	MeSH descriptor: [Weight Reduction Programs] explode all trees
#31	MeSH descriptor: [Diet Therapy] explode all trees
#32	MeSH descriptor: [] explode all trees and with qualifier(s): [diet therapy - DH]
#33	{OR #29-#32}
#34	#28 OR #33
#35	(strateg*):ti OR (strateg*):ab

#36	(trial):ti OR (trial):ab
#37	(random*):ti OR (random*):ab
#38	(placebo):ti OR (placebo):ab
#39	(groups):ti OR (groups):ab
#40	(blind):ti OR (blind):ab
#41	(blinding):ti OR (blinding):ab
#42	(blinded):ti OR (blinded):ab
#43	(control):ti OR (control):ab
#44	(controlled):ti OR (controlled):ab
#45	(rct):ti OR (rct):ab
#46	(intervention):ti OR (intervention):ab
#47	(interventions):ti OR (interventions):ab
#48	(program):ti OR (program):ab
#49	(programs):ti OR (programs):ab
#50	(programme):ti OR (programme):ab
#51	(programmes):ti OR (programmes):ab
#52	(experiment*):ti OR (experiment*):ab
#53	("standard care"):ti OR ("standard care"):ab
#54	("as usual"):ti OR ("as usual"):ab
#55	("follow up"):ti OR ("follow up"):ab
#56	{OR #35-#55}
#57	MeSH descriptor: [Clinical Trial] this term only
#58	MeSH descriptor: [Controlled Clinical Trial] explode all trees
#60	MeSH descriptor: [Validation Study] this term only
#61	MeSH descriptor: [Multicenter Study] this term only
#62	MeSH descriptor: [Evaluation Study] this term only
#63	MeSH descriptor: [Comparative Study] this term only
#64	MeSH descriptor: [Treatment Outcome] this term only
#65	MeSH descriptor: [Clinical Trials as Topic] explode all trees
#66	{OR #57-#65}
#67	#56 OR #66
#68	("body weight"):ti OR ("body weight"):ab
#69	("body composition"):ti OR ("body composition"):ab
#70	("body mass index"):ti OR ("body mass index"):ab
#71	(bmi):ti OR (bmi):ab
#72	("weight gain"):ti OR ("weight gain"):ab
#73	("gain* weight"):ti OR ("gain* weight"):ab
#74	("lose weight"):ti OR ("lose weight"):ab
#75	("lost weight"):ti OR ("lost weight"):ab
#76	("waist circumference"):ti OR ("waist circumference"):ab

#77	(adipos*):ti OR (adipos*):ab
#78	("body fat"):ti OR ("body fat"):ab
#79	(lipid*):ti OR (lipid*):ab
#80	("blood glucose"):ti OR ("blood glucose"):ab
#81	{OR #68-#80}
#82	MeSH descriptor: [Body Mass Index] this term only
#83	MeSH descriptor: [Body Composition] explode all trees
#84	MeSH descriptor: [Weight Gain] this term only
#85	MeSH descriptor: [Body Weight] this term only
#86	MeSH descriptor: [Weight Loss] this term only
#87	MeSH descriptor: [Overweight] explode all trees
#88	MeSH descriptor: [Waist Circumference] explode all trees
#89	MeSH descriptor: [Blood Pressure] this term only
#90	MeSH descriptor: [Lipids] explode all trees
#91	MeSH descriptor: [Blood Glucose] this term only
#92	{OR #82-#91}
#93	#81 OR #92
#94	#15 AND #34 AND #67 AND #93 with Publication Year from 2010 to present, in Trials

Table 6. Inclusion and exclusion criteria using the Participant, Intervention, Comparator, Outcome, Source framework

Component	Inclusion criteria	Exclusion criteria
**Population**	Studies in which the majority of the participants had current clinical diagnosis of major depressive disorder, bipolar and related disorders, schizophrenia spectrum and other psychotic disorders.	Studies featuring participants without clinical diagnosis of serious mental illness.<br>Studies featuring participants with feeding or eating disorders.
**Intervention**	Randomised, non-randomised and cluster randomised interventions with dietary components based on individual or group dietary change programs or individual or group nutrition education.<br>Diet- and nutrition-related interventions embedded within broader lifestyle or health programs.	All other types of studies.
**Comparator**	Studies with ‘treatment as usual’ or ‘standard care’ (e.g., medication and/or other therapy), or inactive (e.g., waitlist) as control conditions.	Studies with active lifestyle-based control conditions.
**Outcome**	Studies with the following primary outcomes: body weight or composition, BMI, blood lipids (cholesterol, HDL, LDL), blood pressure, blood glucose, triglycerides and HbA1c.	Studies with any other primary outcomes.

BMI: body mass index; HbA1c: glycated haemoglobin; HDL: high density lipoprotein; LDL: low density lipoprotein.

Risk of bias assessment

Figure 1. Weighted bar plots for distribution of risk of bias assessment within each bias domain for the two non-randomised studies

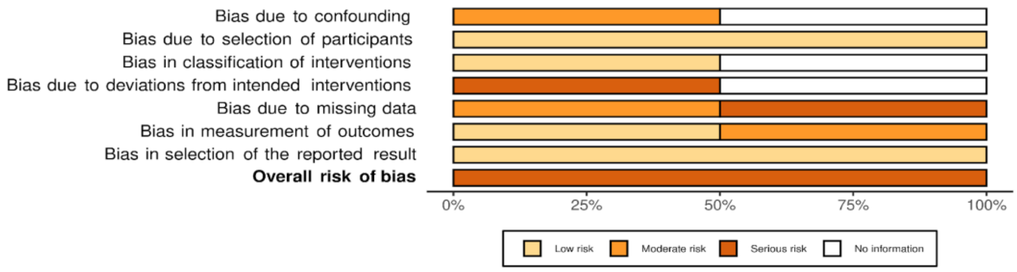

Figure 2. Traffic light plot of the domain-level assessment for the two non-randomised studies

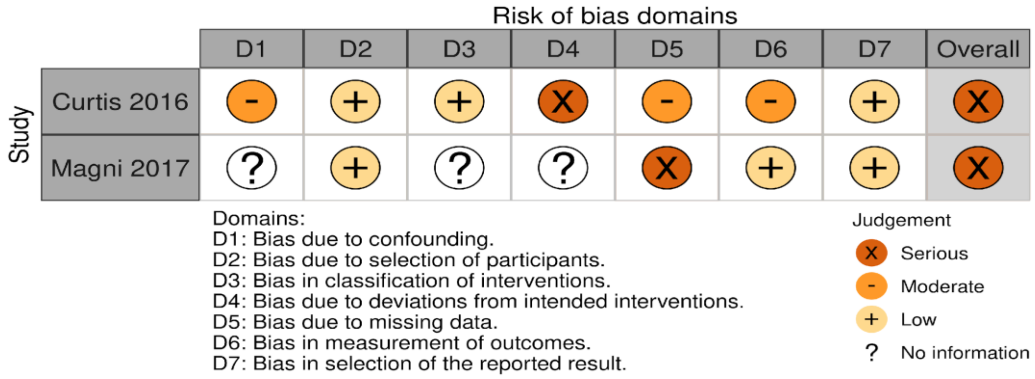

Figure 3. Traffic light plot of the domain-level assessment for the three cluster randomised trials

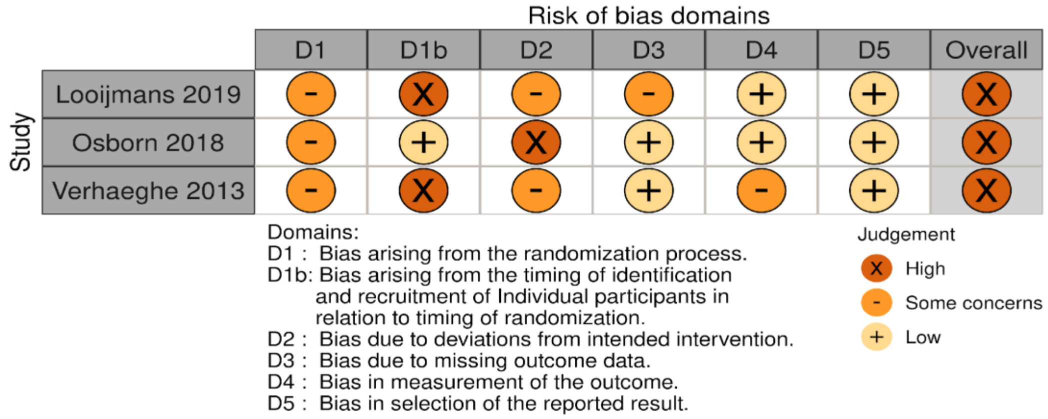

Figure 4. Weighted bar plots for distribution of risk of bias assessment within each bias domain for the twenty randomised controlled trials

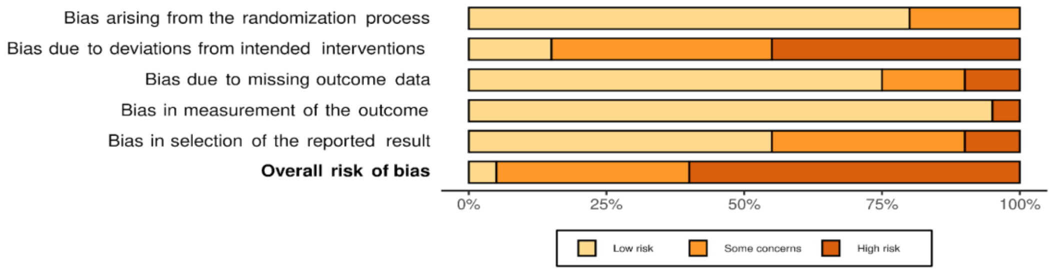

Figure 5. Traffic light plot of the domain-level assessment for the twenty randomised controlled trials

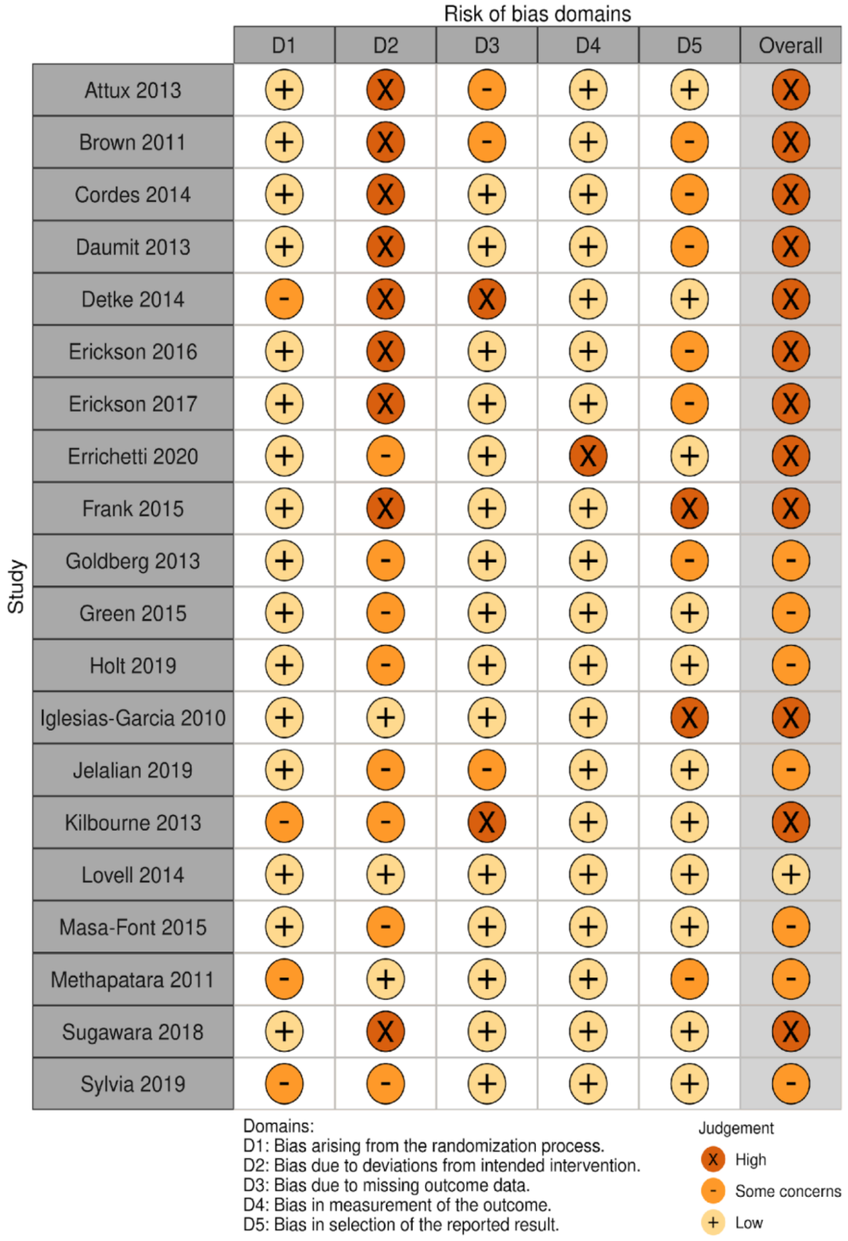

Table 7. Assessment of evidence using Grading of Recommendations Assessment, Development and Evaluation (GRADE)

Outcome	Risk of bias (1)	Inconsistency (2)	Indirectness (3)	Imprecision (4)	GRADE	Interpretation
**Body weight, composition**						
BMI (kg/m^2)	Low	Very low	Moderate	Low	Low	It is uncertain whether dietary interventions change body mass index in people with severe mental illness
Waist circumference (cm)	Low	Low	Moderate	Low	Low	It is uncertain whether dietary interventions change waist circumference in people with severe mental illness
Weight (kg)	Low	Low	Moderate	Low	Low	It is uncertain whether dietary interventions change body weight in people with severe mental illness
**Blood pressure**						
Blood pressure diastolic (mmHg)	Low	Low	Low	Moderate (5)	Low	It is uncertain whether dietary interventions reduce diastolic blood pressure in people with severe mental illness
Blood pressure systolic (mmHg)	Low	Low	Low	Moderate (5)	Low	It is uncertain whether dietary interventions reduce systolic blood pressure in people with severe mental illness
**Blood glucose**						
Glucose (mmol/L)	Low	Moderate (6)	Low	Low	Low	It is uncertain whether dietary interventions reduce blood sugar levels in people with severe mental illness
HbA1c (mmol/mol)	Low	Moderate (6)	Low	Moderate (5)	Low	It is uncertain whether dietary interventions reduce HbA_{1c} in people with severe mental illness
**Blood lipids**						
HDL-cholesterol (mmol/L)	Low	Low	Very low	Low	Low	It is uncertain whether dietary interventions improve HDL levels in people with severe mental illness
LDL-cholesterol (mmol/L)	Low	Low	Very low	Very low (7)	Very low (7)	It is uncertain whether dietary interventions improve LDL levels in people with severe mental illness
Total cholesterol (mmol/L)	Low	Moderate (6)	Very low	Low	Low	It is uncertain whether dietary interventions reduce total cholesterol levels in people with severe mental illness
Triglycerides (mmol/L)	Low	Moderate (6)	Very low	Low	Low	It is uncertain whether dietary interventions reduce triglyceride levels in people with severe mental illness

BMI: body mass index; HbA1c: glycated haemoglobin; HDL: high density lipoprotein; LDL: low density lipoprotein.

(1) Downgraded for high risk of bias as assessed by ROB2, ROB2 Cluster, ROBINS-I
(2) Downgraded for inconsistency: heterogeneity (I^2) (downgraded further if high heterogeneity), differences in PICO, intervention type and duration
(3) Downgraded as some studies were not designed to assess this outcome as a primary outcome (downgraded further if the majority of studies were not designed to assess this outcome)
(4) Downgraded as some studies had a small sample size and hence wide confidence intervals, dietary intervention design and adherence varied introducing imprecision
(5) Downgraded due to variation in dietary intervention design and adherence
(6) Downgraded due to differences in PICO, intervention type and duration
(7) Downgraded for imprecision due to small number of studies/ fewer participants.

Figure 6. Subgroup analysis: Forest plot for the impact of diet/nutrition-based interventions in which the nutrition component was delivered by a nutrition professional (eg, dietitian) on weight

Study or Subgroup	Experimental Mean	SD	Total	Control Mean	SD	Total	Weight	Std. Mean Difference IV, Random, 95% CI
Attux 2013	80.7	14.1	81	84.7	17.9	79	26.9%	−0.25 [−0.56, 0.06]
Brown 2011	99.4	18.5	68	109.7	27.5	68	24.6%	−0.44 [−0.78, −0.10]
Cordes 2014	81.9	15.1	36	79	15	38	17.4%	0.19 [−0.27, 0.65]
Curtis 2016	69.9	13.9	16	83.6	19	12	7.7%	−0.82 [−1.60, −0.03]
Goldberg 2013	109.23	18.19	53	103.46	19.69	56	0.0%	0.30 [−0.08, 0.68]
Holt 2019	104.1	21.1	207	101.3	23.7	205	0.0%	0.12 [−0.07, 0.32]
Iglesias-Garcia 2010	92.3	8.6	7	95.3	22.7	7	0.0%	−0.16 [−1.21, 0.89]
Sugawara 2019a	79.5	11.8	67	82.4	11.7	61	0.0%	−0.25 [−0.59, 0.10]
Sugawara 2019b	77.9	16.3	61	82.4	11.7	61	23.4%	−0.32 [−0.67, 0.04]
Sylvia 2019	90.31	16.6	13	102.51	25.85	12	0.0%	−0.55 [−1.35, 0.25]
Verhaeghe 2013	87.6	20.77	201	85.41	16.49	83	0.0%	0.11 [−0.14, 0.37]
**Total (95% CI)**			**262**			**258**	**100.0%**	**−0.28 [−0.51, −0.04]**

Heterogeneity: $Tau^2 = 0.03$; $Chi^2 = 6.79$, df = 4 (P = 0.15); $I^2 = 41\%$
Test for overall effect: Z = 2.30 (P = 0.02)

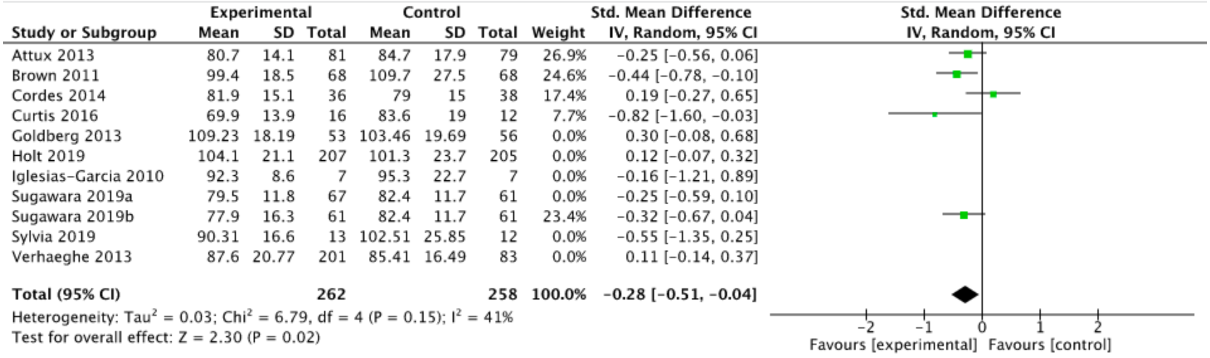

CI: confidence interval; df: degrees of freedom; SD: standard deviation; Std: standardised.

Figure 7. Subgroup analysis: Forest plot of trials that examined the impact of diet/nutrition-based interventions on body weight (kg) in which the nutrition component was delivered individually

Study or Subgroup	Experimental Mean	SD	Total	Control Mean	SD	Total	Weight	Std. Mean Difference IV, Random, 95% CI
Attux 2013	80.7	14.1	81	84.7	17.9	79	0.0%	−0.25 [−0.56, 0.06]
Brown 2011	99.4	18.5	68	109.7	27.5	68	0.0%	−0.44 [−0.78, −0.10]
Cordes 2014	81.9	15.1	36	79	15	38	0.0%	0.19 [−0.27, 0.65]
Curtis 2016	69.9	13.9	16	83.6	19	12	0.0%	−0.82 [−1.60, −0.03]
Goldberg 2013	109.23	18.19	53	103.46	19.69	56	0.0%	0.30 [−0.08, 0.68]
Holt 2019	104.1	21.1	207	101.3	23.7	205	0.0%	0.12 [−0.07, 0.32]
Iglesias-Garcia 2010	92.3	8.6	7	95.3	22.7	7	0.0%	−0.16 [−1.21, 0.89]
Sugawara 2019a	79.5	11.8	67	82.4	11.7	61	46.8%	−0.25 [−0.59, 0.10]
Sugawara 2019b	77.9	16.3	61	82.4	11.7	61	44.4%	−0.32 [−0.67, 0.04]
Sylvia 2019	90.31	16.6	13	102.51	25.85	12	8.8%	−0.55 [−1.35, 0.25]
Verhaeghe 2013	87.6	20.77	201	85.41	16.49	83	0.0%	0.11 [−0.14, 0.37]
**Total (95% CI)**			**141**			**134**	**100.0%**	**−0.30 [−0.54, −0.06]**

Heterogeneity: $Tau^2 = 0.00$; $Chi^2 = 0.47$, df = 2 (P = 0.79); $I^2 = 0\%$
Test for overall effect: Z = 2.49 (P = 0.01)

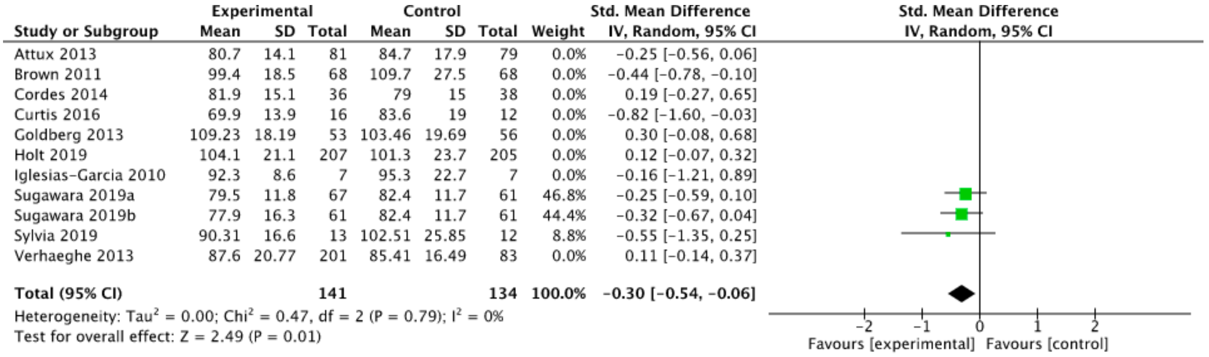

CI: confidence interval; df: degrees of freedom; SD: standard deviation; Std: standardised.

Figure 8. Funnel plot of the meta-analysis of the included studies that investigated the impact of diet/nutrition-based interventions on body weight

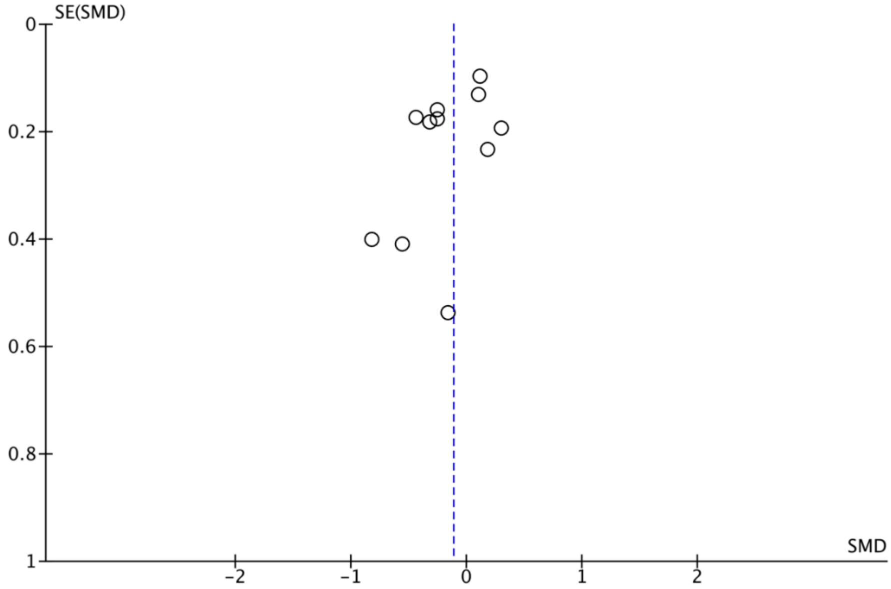

SE: standard error; SMD: standardised mean difference.

Figure 9. Subgroup analyses: Forest plots for the impact of diet/nutrition-based interventions in which the nutrition component was delivered by a nutrition professional (e.g., dietitian) on blood pressure.

A

	Experimental			Control				Std. Mean Difference
**Study or Subgroup**	**Mean**	**SD**	**Total**	**Mean**	**SD**	**Total**	**Weight**	**IV, Random, 95% CI**
Attux 2013	114.1	12.2	81	115.6	12.4	79	23.5%	−0.12 [−0.43, 0.19]
Errichetti 2020	117.3	17.7	249	120.3	17.5	167	58.7%	−0.17 [−0.37, 0.03]
Holt 2019	125	15	207	122	16	205	0.0%	0.19 [−0.00, 0.39]
Kilbourne 2013	127.7	17.7	57	134.2	18.9	59	0.0%	−0.35 [−0.72, 0.01]
Masa-Font 2015	123.13	16.82	169	123.54	18.49	163	0.0%	−0.02 [−0.24, 0.19]
Osborn 2018	125	16	155	126	17	172	0.0%	−0.06 [−0.28, 0.16]
Sugawara 2019a	126.9	14.7	67	128.1	15	61	0.0%	−0.08 [−0.43, 0.27]
Sugawara 2019b	123.3	15.1	61	128.1	15	61	17.7%	−0.32 [−0.67, 0.04]
**Total (95% CI)**			**391**			**307**	**100.0%**	**−0.18 [−0.34, −0.03]**

Heterogeneity: $Tau^2 = 0.00$; $Chi^2 = 0.71$, df = 2 (P = 0.70); $I^2 = 0\%$
Test for overall effect: Z = 2.40 (P = 0.02)

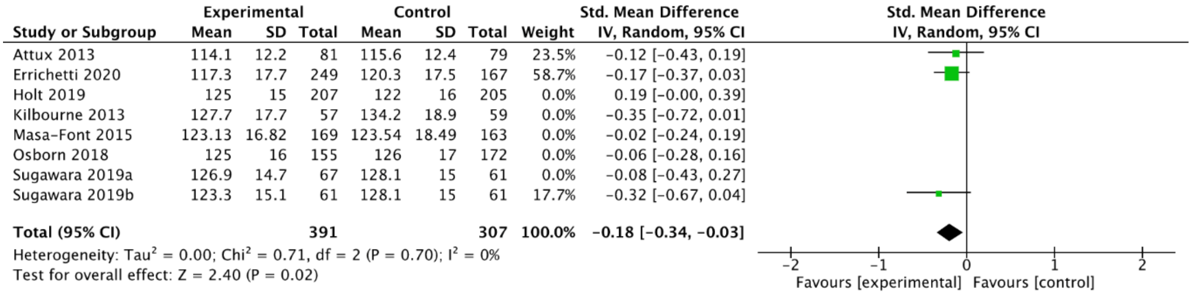

B

	Experimental			Control				Std. Mean Difference
**Study or Subgroup**	**Mean**	**SD**	**Total**	**Mean**	**SD**	**Total**	**Weight**	**IV, Random, 95% CI**
Attux 2013	75.9	10.3	81	78.8	10.9	79	23.4%	−0.27 [−0.58, 0.04]
Errichetti 2020	73.4	10.9	249	74.6	10.5	167	58.8%	−0.11 [−0.31, 0.08]
Holt 2019	82	10	207	81	11	205	0.0%	0.09 [−0.10, 0.29]
Kilbourne 2013	75.3	10.6	57	80.5	10.3	59	0.0%	−0.49 [−0.86, −0.12]
Masa-Font 2015	80.47	10.52	169	79.71	11.11	163	0.0%	0.07 [−0.15, 0.29]
Osborn 2018	80	10	155	80	10	172	0.0%	0.00 [−0.22, 0.22]
Sugawara 2019a	78.9	14	67	82.1	12.1	61	0.0%	−0.24 [−0.59, 0.11]
Sugawara 2019b	79.1	11	61	82.1	12.1	61	17.8%	−0.26 [−0.61, 0.10]
**Total (95% CI)**			**391**			**307**	**100.0%**	**−0.18 [−0.33, −0.02]**

Heterogeneity: $Tau^2 = 0.00$; $Chi^2 = 0.98$, df = 2 (P = 0.61); $I^2 = 0\%$
Test for overall effect: Z = 2.28 (P = 0.02)

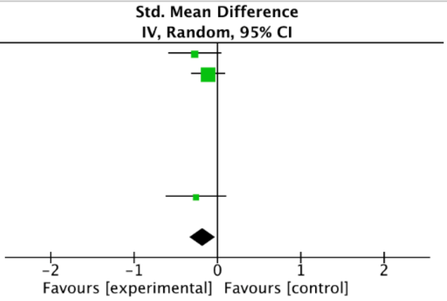

A) systolic blood pressure; B) diastolic blood pressure. CI: confidence interval; df: degrees of freedom; SD: standard deviation; Std: standardised.
